# Two-year antibody persistence in children vaccinated at 12–15 months with a measles-mumps-rubella virus vaccine without human serum albumin

**DOI:** 10.1080/21645515.2017.1309486

**Published:** 2017-05-08

**Authors:** Andrea A. Berry, Remon Abu-Elyazeed, Clemente Diaz-Perez, Maurice A. Mufson, Christopher J. Harrison, Michael Leonardi, Jerry D. Twiggs, Christopher Peltier, Stanley Grogg, Antonio Carbayo, Steven Shapiro, Michael Povey, Carmen Baccarini, Bruce L. Innis, Ouzama Henry

**Affiliations:** a Center for Vaccine Development, Institute for Global Health, University of Maryland School of Medicine, Baltimore, MD, USA; b GSK, Philadelphia, PA, USA; c School of Medicine, Medical Sciences Campus, University of Puerto Rico, San Juan PR, Puerto Rico; d Joan C. Edwards School of Medicine, Marshall University, Huntington, WV, USA; e Children's Mercy Hospital and Clinics, and University of Missouri at Kansas City, Kansas City, MO, USA; f Palmetto Pediatrics PA, North Charleston, SC, USA; g Dixie Pediatrics, Saint George, UT, USA; h Pediatric Associates of Mount Carmel, Inc., Cincinnati, OH, USA; i Oklahoma State University, Center for Health Sciences, Tulsa, OK, USA; j Full Health University Medical Clinic, Santa Ana, CA, USA; k Pediatric Medical Associates, Norriton, PA, USA; l GSK, Wavre, Belgium; m GSK, King of Prussia, PA, USA

**Keywords:** antibody persistence, MMR vaccine, measles, mumps, *Priorix*, rubella

## Abstract

One combined measles-mumps-rubella (MMR) vaccine without Human Serum Albumin (HSA) is currently licensed in the USA (M-M-R II; Merck, USA) and another has been developed (*Priorix*™ [MMR-RIT, GSK, Belgium]). In this follow-up study, children from USA or Puerto Rico, who had received one dose of M-M-R II or MMR-RIT at 12–15 months of age in the primary study (NCT00861744), were followed-up for 2 y post-vaccination. Anti-measles and anti-rubella antibodies were measured using Enzyme-Linked Immunosorbent Assay (ELISA), and anti-mumps antibodies using ELISA and plaque reduction neutralization (PRN) assays. Serious adverse events (SAEs) were recorded during the entire follow-up. The according-to-protocol (ATP) persistence cohort included 752 children (M-M-R II = 186, MMR-RIT = 566), who received primary vaccination at a mean age of 12.3 ( ± 0.67) months. 104 children were revaccinated with MMR-containing vaccines; therefore, serology results for timepoints after revaccination were excluded from the analysis. Seropositivity for measles (Year 1≥ 98.3%; Year 2≥ 99.4%) and rubella (Year 1≥ 98.9%; Year 2 = 100%) remained as high at Year 2 as at Day 42. Similarly, seropositivity for mumps determined by ELISA (Year 1≥ 90.1%; Year 2≥ 94.1%) and PRN assays (Year 1≥ 87.5%; Year 2≥ 91.7%) persisted. Thirty-three SAEs were recorded in 23 children; 2 SAEs (inguinal adenitis and idiopathic thrombocytopenic purpura) and one SAE (febrile convulsion) were considered as potentially related to MMR-RIT and M-M-R II, respectively. This study showed that antibodies against measles, mumps and rubella persisted for up to 2 y post-vaccination with either MMR vaccine in children aged 12–15 months, and that both vaccines were well-tolerated during the follow-up period.

## Introduction

Measles, mumps and rubella (MMR) combined vaccine, which has been available in the United States of America (USA) since 1971,^1^ has greatly reduced the incidence of these diseases.^2^ Nevertheless, attaining high vaccine coverage and maintaining these levels are essential in the prevention and elimination of these childhood infectious illnesses.^3,4^

Although the vaccine coverage for at least one dose of MMR vaccine was 91.5% in 2014, one in every 12 children in the USA did not receive their first dose of vaccine on time, causing high measles susceptibility in some locations.^5^ Reasons for low coverage of measles vaccine in certain areas could be due to vaccine hesitancy and lack of access to care,^5^ or intentional non-vaccination due to personal beliefs.^6^

Measles outbreaks occur predominantly in unvaccinated individuals, and are facilitated by low coverage as well as the high transmissibility of the measles virus.^7,8^ As a result of sub-optimal vaccine uptake, the USA has experienced several recent measles outbreaks.^8^ In 2014 and 2015, 667 and 189 cases of measles were reported to the Center for Disease Control (CDC), respectively, and in 2016, a preliminary count of 70 measles cases were reported in the USA.^6,8,9^ The largest outbreak in 2015 originated at an amusement park in California, and largely affected unvaccinated children.^6^ In 2014, a single large outbreak affecting 383 cases occurred primarily among unvaccinated Amish communities in Ohio. Many cases in 2014 were associated with people migrating from the Philippines, where there had been a large outbreak of measles.^6,10,11^

MMR vaccination is recommended in over 100 countries, including the European Union, North America and Australasia.^12^ In the USA, the Advisory Committee on Immunization Practices recommends 2 doses of MMR vaccine for children, with the first dose administered at 12−15 months of age followed by a second dose usually given before school entry in children aged 4 to 6 y (the second dose can be administered any time, with a minimum of 28 d between the doses).^3^ Currently there is only one MMR vaccine licensed in the USA (M-M-R II; Merck, USA), and any interruption to this single supply line could be a public health risk.^3^ Another MMR vaccine, *Priorix™* (MMR-RIT [RIT strain 4385]; GSK, Belgium), is licensed in over 100 countries^13^ and has been shown to be immunogenic and well-tolerated in trials conducted in the USA.^13-15^ Furthermore, as the MMR-RIT vaccine, like the current formulation of the M-M-R II vaccine, is manufactured without Human Serum Albumin (HSA) in accordance with the European Medicines Agency (EMA) guidelines, the theoretical risk of microbial contamination is reduced compared with previous formulations of the M-M-R II vaccine.^16,17^

We described previously the short-term antibody responses to first doses of MMR-RIT and M-M-R II administered to healthy US children between 12–15 months of age;^13^ in this article, we report the antibody persistence at one and 2 y post-vaccination.

## Results

### Demographic data

**Figure 1.**
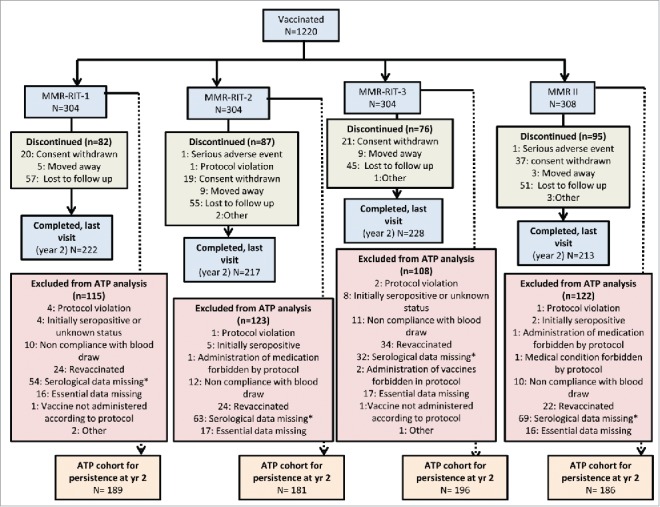
Disposition of participants in the total vaccinated cohort (persistence phase). ATP, according-to-protocol; MMR: Measles Mumps Rubella vaccine. *For children revaccinated at any point in the study, data were censored from analysis for timepoints after revaccination.

In the primary phase of the study, 1220 children received a single dose of either one of 3 MMR-RIT lots, each containing different RIT 4385 mumps strain titers: high (10^4.8^ CCID_50_; MMR-RIT-1 group), medium (10^4.1^ CCID_50_; MMR-RIT-2 group) or low (10^3.7^ CCID_50_; MMR-RIT-3 group), or the M-M-R II vaccine (M-M-R II group). Of these, 880 children completed the 2-year persistence phase (Fig. 1). The according-to-protocol (ATP) cohort for the persistence phase included 752 children, who had no exclusion criteria for the study, had not received a vaccine forbidden in the protocol, complied with blood sampling schedules, and for whom immunogenicity end point measures were available for pre-vaccination, Day 42 and Year 2 post-vaccination.

The ATP persistence cohort had a mean age at primary vaccination of 12.3 (standard deviation: ± 0.6) months; 78.1% of children were White/Caucasian and 51.6% were male. The demographic characteristics were similar among the 4 treatment groups (Table 1).

**Table 1.** Demographic characteristics (ATP cohort for persistence).

Characteristics	Categories	MMR-RIT-1N = 189		MMR-RIT-2 N = 181		MMR-RIT-3 N = 196		M-M-R IIN = 186		
Age at dose 1	Mean	12.3		12.3		12.2		12.3		
(months)	SD	0.67		0.66		0.55		0.67		
		n	%	n	%	n	%	n	%	
Gender	Female	98	51.9	87	48.1	100	51.0	79	42.5	
	Male	91	48.1	94	51.9	96	49.0	107	57.5	
Race	African heritage	14	7.4	15	8.3	16	8.2	18	9.7	
	American Indian or Alaskan native	0	0.0	0	0.0	0	0.0	1	0.5	
	South East Asian heritage	1	0.5	1	0.6	0	0.0	0	0.0	
	Pacific islander	2	1.1	0	0.0	1	0.5	2	1.1	
	North African heritage	2	1.1	0	0.0	1	0.5	3	1.6	
	European heritage	150	79.4	140	77.3	154	78.6	143	76.9	
	Other	20	10.6	25	13.8	24	12.2	19	10.2	

MMR-RIT-1: lot 1 of MMR

MMR-RIT-2: lot 2 of MMR

MMR-RIT-3: lot 3 of MMR

M-M-R II: Merck's MMR vaccine

N = total number of children

SD: Standard deviation

n/% = number / percentage of children in a given category

In this study, a total of 104 (8.5%) children were revaccinated (MMR-RIT-1 [24/304; 7.9%]; MMR-RIT-2 [24/304; 7.9%]; MMR-RIT-3 [34/304; 11.2%]; M-M-R II [22/308; 7.1%]) after the visit at Day 42 (86 children) or Year 1 (18 children). Among the 104 revaccinated children, 84 received the additional MMR-containing vaccine dose (either M-M-R II or *ProQuad*® [Merck, USA]) after being identified as sub-optimal responders for at least one antigen at Day 42, and 20 were revaccinated for other reasons; their serology results for timepoints after revaccination were excluded from the analysis. There were an additional 19 sub-optimal responders identified at Day 42 who were not revaccinated and therefore were included in the analyses.

### Immunogenicity

#### Measles

Seropositivity rates for anti-measles antibodies were 98.3–100% across the 3 MMR-RIT groups and 99.4% in the M-M-R II group at Year 1, and 99.4–100% across the 3 MMR-RIT groups and 100% in the M-M-R II group at Year 2.

Anti-measles antibody geometric mean concentrations (GMCs) persisted through Years 1 and 2, and remained at least as high as those recorded at Day 42; the ranges were 3230.2−4022.1 mIU/mL across the 4 groups (Table 2). These antibody responses against the measles virus were at least 16-fold higher than the seroresponse threshold at Years 1 and 2. Among the 10 children who had sub-optimal measles responses at Day 42 and were revaccinated during the 2-year period following vaccination, 8 had post-revaccination titers above the threshold for measles and 2 did not have post-revaccination blood samples.

**Table 2.** Percentage of children with anti-measles, mumps and rubella antibodies in initially seronegative children (ATP cohort for persistence).

Group	Timing	N	n	% (95% CI)	GMC (95% CI)
Anti-measles antibody ≥200 mIU/mL					
MMR-RIT-1	D42	182	181	99.5 (97.0−100)	2779.3 (2509.9−3077.7)
	Y1	179	178	99.4 (96.9−100)	3230.2 (2820.4−3699.7)
	Y2	171	171	100 (97.9−100)	3361.1 (2922.3−3865.6)
MMR-RIT-2	D42	172	169	98.3 (95.0−99.6)	3010.1 (2669.0−3394.7)
	Y1	175	172	98.3 (95.1−99.6)	3766.9 (3245.6−4372.0)
	Y2	159	159	100 (97.7−100)	3963.8 (3479.3−4515.7)
MMR-RIT-3	D42	186	184	98.9 (96.2−99.9)	2703.0 (2446.6−2986.3)
	Y1	191	191	100 (98.1−100)	3521.5 (3094.6−4007.3)
	Y2	169	168	99.4 (96.7−100)	3360.3 (2923.3−3862.7)
M-M-R II	D42	178	177	99.4 (96.9−100)	2749.6 (2469.9−3061.0)
	Y1	178	177	99.4 (96.9−100)	3930.4 (3423.3−4512.7)
	Y2	166	166	100 (97.8−100)	4022.1 (3507.7−4611.9)
Anti-mumps (PPD ELISA) ≥10 ELU/mL					
MMR-RIT-1	D42	159	152	95.6 (91.1−98.2)	56.8 (48.7−66.3)
	Y1	141	127	90.1 (83.9−94.5)	47.0 (37.9−58.2)
	Y2	136	128	94.1 (88.7−97.4)	47.8 (40.2−56.9)
MMR-RIT-2	D42	153	142	92.8 (87.5−96.4)	43.4 (37.3−50.4)
	Y1	142	129	90.8 (84.9−95.0)	40.1 (33.4−48.0)
	Y2	130	125	96.2 (91.3−98.7)	50.2 (42.1−59.9)
MMR-RIT-3	D42	169	153	90.5 (85.1−94.5)	46.1 (39.5−53.8)
	Y1	154	139	90.3 (84.4−94.4)	43.9 (36.5−52.9)
	Y2	141	136	96.5 (91.9−98.8)	54.0 (46.1−63.3)
M-M-R II	D42	158	148	93.7 (88.7−96.9)	55.3 (47.9−63.8)
	Y1	146	140	95.9 (91.3−98.5)	57.4 (49.1−67.0)
	Y2	140	134	95.7 (90.9−98.4)	59.2 (50.1−70.0)
Anti-mumps (unenhanced PRN assay) ^*^≥ 4 ED_50_					
MMR-RIT-1	D42	71	53	74.6 (62.9−84.2)	11.8 (8.4−16.5)
	Y1	161	142	88.2 (82.2−92.7)	33.1 (24.9−44.2)
	Y2	157	144	91.7 (86.3−95.5)	43.4 (33.4−56.3)
MMR-RIT-2	D42	69	55	79.7 (68.3−88.4)	17.3 (11.8−25.4)
	Y1	170	152	89.4 (83.8−93.6)	40.2 (31.0−52.1)
	Y2	144	134	93.1 (87.6−96.6)	48.9 (37.7−63.5)
MMR-RIT-3	D42	73	61	83.6 (73.0−91.2)	16.0 (11.4−22.4)
	Y1	184	161	87.5 (81.8−91.9)	42.7 (32.9−55.4)
	Y2	157	152	96.8 (92.7−99.0)	57.4 (45.7−72.2)
M-M-R II	D42	82	59	72.0 (60.9−81.3)	14.3 (10.3−19.9)
	Y1	167	148	88.6 (82.8−93.0)	46.4 (35.7−60.3)
	Y2	152	144	94.7 (89.9−97.7)	60.7 (47.6−77.5)
Anti-rubella antibody ≥10 IU/mL					
MMR-RIT-1	D42	182	180	98.9 (96.1−99.9)	74.3 (66.4−83.1)
	Y1	179	177	98.9 (96.0−99.9)	136.4 (121.4−153.3)
	Y2	171	171	100 (97.9−100)	78.0 (69.7−87.2)
MMR-RIT-2	D42	170	167	98.2 (94.9−99.6)	74.4 (65.8−84.0)
	Y1	174	173	99.4 (96.8−100)	134.8 (121.4−149.8)
	Y2	158	158	100 (97.7−100)	79.5 (71.7−88.2)
MMR-RIT-3	D42	185	182	98.4 (95.3−99.7)	67.8 (61.0−75.5)
	Y1	190	189	99.5 (97.1−100)	135.6 (122.0−150.7)
	Y2	168	168	100 (97.8−100)	81.7 (73.8−90.4)
M-M-R II	D42	178	178	100 (97.9−100)	89.6 (80.3−100.0)
	Y1	178	178	100 (97.9−100)	165.7 (149.4−183.9)
	Y2	166	166	100 (97.8−100)	93.1 (83.6−103.6)

MMR-RIT-1: lot 1 of MMR

MMR-RIT-2: lot 2 of MMR

MMR-RIT-3: lot 3 of MMR

M-M-R II: Merck's MMR vaccine

D42= Post-vaccination blood sample at Day 42

Y1= Antibody persistence blood sample at Year 1

Y2= Antibody persistence blood sample at Year 2

N = number of subjects with available results; n/% = number/percentage of children with concentration above the specified value; 95% CI = 95% confidence interval

GMC= geometric mean antibody concentration calculated on all children

* Enhanced PRN assay was used in the first year and unenhanced PRN assay was used in the second year

#### Mumps

Using the PPD ELISA, seropositivity rates were 90.1–90.8% across the 3 MMR-RIT groups and 95.9% for the M-M-R II group at Year 1, and 94.1–96.5% across the 3 MMR-RIT groups and 95.7% for the M-M-R II group at Year 2. The corresponding GMCs at Years 1 and 2 across the 4 groups were at least 4-fold higher than the seroresponse threshold (Table 2).

Using the unenhanced-PRN assay, seropositivity rates were 87.5–89.4% across the 3 MMR-RIT groups and 88.6% in the M-M-R II group at Year 1, and 91.7–96.8% across the 3 MMR-RIT groups and 94.7% in the M-M-R II group at Year 2. The corresponding geometric mean titers (GMTs) across the 4 groups at Years 1 and 2 were also observed to be at least 8-fold higher than the seroresponse threshold (Table 2).

Sixty-six of 72 (91.7%) children who were revaccinated during the 2-year period following vaccination due to sub-optimal response for mumps at Day 42 (based on the enhanced-plaque reduction neutralization [PRN] assay) were above the thresholds for mumps PRN (using unenhanced-PRN assay) or purified protein derivative (PPD) enzyme-linked immunosorbent assay (ELISA) following revaccination, 4/72 (5.6%) had no post-revaccination blood samples, and 2/72 (2.8%) were below the seroresponse threshold of the mumps PPD ELISA following revaccination.

#### Rubella

The seroresponse for rubella was similar in all 4 vaccine groups, ranging from 98.9–99.5% for the 3 MMR-RIT groups and reaching 100% for the M-M-R II group at Year 1, while at Year 2, all children were seropositive in the 4 groups (Table 2). The corresponding GMCs were higher at Year 1 (range: 134.8−165.7 IU/mL) than at pre-vaccination, but declined at Year 2 (range: 78.0−93.1 IU/mL) across the 4 groups. These antibody responses against rubella were at least 8-fold higher than the seroresponse threshold at Years 1 and 2 (Table 2). Among the 8 children who had sub-optimal rubella responses at Day 42 and were revaccinated during the 2-year period following vaccination, 7 were above the thresholds for rubella following revaccination, and one did not have post-revaccination blood samples.

### Safety

During the extended safety follow-up phase (up to 6 months), 32 serious adverse events (SAEs) were reported in 22 children: MMR-RIT-1 (n = 1), MMR-RIT-2 (n = 6), MMR-RIT-3 (n = 7), M-M-R II (n = 8). Three SAEs were considered by the investigators as potentially related to the study vaccine: one case of inguinal adenitis at Day 68 in the MMR-RIT-1 group, which resolved within 14 days; one case of grade 2 idiopathic thrombocytopenic purpura with onset at Day 20 in the MMR-RIT-2 group, which resolved within 212 days; and one case of grade 2 febrile convulsion on Day 0 in the M-M-R II group, which resolved within one day. All SAEs resolved and no fatal SAEs were reported up to 6 months. New onset chronic illness (NOCI) were reported in 13 children: MMR-RIT-1 (n = 5), MMR-RIT-2 (n = 2), MMR-RIT-3 (n = 4), M-M-R II (n = 2).

During the persistence phase (up to 2 y post-vaccination), one child developed nephroblastoma after vaccination with M-M-R II and was withdrawn from the study. This event was not causally related to vaccination as deemed by the investigator. No children experienced SAEs related to study participation, and no deaths occurred during the study.

## Discussion

Our results indicate that protective immune responses to measles, mumps, and rubella observed immediately after administration of a single dose of any of the 3 MMR-RIT lots (containing different mumps virus titers) or M-M-R II persisted for at least 2 y post-vaccination. The immune responses observed in this persistence study were at least as high as the primary responses observed at 42 d post-vaccination, with antibody GMCs/GMTs at Years 1 and 2 remaining at least 4-fold higher than the seroresponse thresholds. These persistence data indicate that protection against the 3 viruses is likely to be maintained between the first dose given at 12–15 months of age and the second dose administered at 4−6 y of age.

Our observations with respect to the persistence of measles and rubella antibodies are consistent with previous findings with the MMR-RIT vaccine.^18-20^ Dine *et al.* showed that antibodies to measles persist for up to 26–33 y after a 2-dose vaccination schedule (dose 1 administered predominantly during the first year of life and revaccination 1−7 y thereafter).^21^ In the present study, we observed that mumps neutralizing antibody titers and corresponding antibody GMTs were either maintained or steadily rose over time, which has also been noted previously.^22,23^ As in our study, these previous publications reported low neutralization titers immediately post-vaccination that then rose in the long-term follow-up period, suggesting that the development of neutralizing antibody titers is rather slow after vaccination. In contrast, ELISA proved to be more sensitive in detecting mumps antibodies soon after vaccination, with only a small increase in the late post-vaccination period.^22,23^ Although rubella seroconversion was not lost over time, we observed an increase in rubella antibodies at Year 1 with a subsequent decline at Year 2 post-primary vaccination. Nevertheless, the Year 2 antibody GMCs were comparable to those observed at Day 42 post-vaccination, suggesting that immunity persists for at least 2 y. The lower response observed at Day 42 could be because the incubation period for the wild type rubella virus replication is up to 21 days, suggesting that the development of the full antibody response could take longer than 42 d.^24^

A secondary, yet noteworthy aspect of our study is the use of 2 different assays to analyze the mumps titers and seroresponse over 2 y. Although there is no proven correlate of protection for mumps, functional assays, such as the PRN assay, are probably a better estimate compared with ELISA because neutralization is a functional aspect of antibodies, whereas ELISA measures total antibodies whether functional or not. In this study, the unenhanced PRN assay yielded seroresponse rates >70% at Day 42 post-vaccination, which is consistent with effectiveness studies following single dose vaccination.^25^ We saw a more pronounced rise in antibody titers over time with PRN assay than with ELISA, likely because antibody levels as measured by PRN continued to increase after vaccination. Recently, Latner *et al.* measured mumps antibody levels using both PRN assay and ELISA specific for the mumps nucleoprotein and hemagglutinin.^26^ They proposed that the differences in the response to the individual mumps proteins could partially explain the lack of correlation between the different serological tests.^26^ The data further indicated that some individuals who were seropositive by ELISA had low levels of neutralizing antibodies, suggesting that previous estimates of immunity based on whole virus ELISA may be overstated.^26^

We studied the persistence of antibodies against measles, mumps and rubella in the context of an investigational MMR vaccine (without HSA), co-administered with existing standard of care vaccines; this is a major strength of this study. The persistence data reflect antibody GMT/GMC values at 12 and 24 months after the first dose of MMR vaccine, which were measured in children aged 2 and 3 y, before the second dose administration, which is usually scheduled at 4 to 6 y of age. The functional antibody assays used in this study to evaluate the persistence of the immune response to mumps are important, as there is currently no proven correlate of protection. We also evaluated persistence in a non-endemic setting, where children would have limited ongoing exposure to such viruses, suggesting that the observed antibody responses represent true vaccine antibody persistence.

It is important to note that since this antibody persistence analysis was a secondary analysis of the overall study, the results are only descriptive. Another limitation of this study was that a small group of children (8.5%) were revaccinated, and their data were censored from this analysis for timepoints after revaccination. As these children received a second dose of MMR during the 2-year period following vaccination, inclusion of these children would have overestimated antibody persistence following one dose of MMR vaccine. The requirement to switch the mumps PRN assay during the study was also a limitation; however, since the old PRN assay used in the first year is known to overestimate titers and likely overestimate protection,^27^ the use of the new PRN assay can be perceived as a strength.

In conclusion, antibodies against measles, mumps and rubella viruses persisted for up to 2 y after primary vaccination at 12–15 months of age with MMR-RIT and M-M-R II vaccines in healthy children. Serological results were comparable for the USA licensed comparator and the investigational MMR-RIT vaccine, and both vaccines were well tolerated during the 2-y post-vaccination follow-up.

## Methods

### Study design

The initial phase II, randomized, observer-blind study was conducted between June 2009 and July 2010 at 48 centers in the USA and 3 centers in Puerto Rico (NCT00861744).^13^ In this study, we continued to follow children for 2 y post-primary vaccination. In the primary phase,^13^ 12−15 month-old children who had not been previously immunized against (and had no previous history of) measles, mumps, rubella, varicella and hepatitis A, and had received 3 doses of 7-valent pneumococcal conjugate vaccine (PCV7) within the first year of life (third dose administered ≥ 30 d before enrollment) were enrolled. Exclusion criteria are listed in the previous publication.^13^

In the primary vaccination study, children were randomized into 4 treatment groups and received single doses of either one of 3 MMR-RIT lots (MMR-RIT-1, MMR-RIT-2 or MMR-RIT-3) or M-M-R II.^3^ Children also received concomitant single doses of hepatitis A vaccine (HAV), varicella vaccine (VAR), and the fourth dose of PCV7.

This study was conducted in accordance with the Declaration of Helsinki and Good Clinical Practice guidelines, and each local site was approved by a national, regional, or investigational center institutional review board or independent ethics committee. Written informed consent was obtained from parents/guardians before enrollment.

### Immunogenicity assessment

Blood samples were collected at Year 1 and Year 2 post-primary vaccination. Sera were stored at −20°C until assayed in a blinded manner at a central laboratory (GSK, Rixensart, Belgium).

Immunoglobulin (IgG) antibodies to measles and rubella were measured using a commercial ELISA, *Enzygnost*™ (Dade Behring, Marburg GmbH, Germany). The test was performed and interpreted as directed by the manufacturer. The complement and IgG enhanced-PRN assay used to assess mumps seropositivity in the first year was replaced with a PRN assay without complement and without anti-immunoglobulin enhancement (unenhanced-PRN; using the wild-type virus, MU-90)^27^ to assess the production of neutralizing antibodies. In addition, IgG antibodies to the mumps virus were measured using a quantitative PPD-ELISA (Merck, USA). The replacement of the enhanced-PRN assay with the 2 new assays for the assessment of mumps seropositivity was performed in accordance with the guidance from the Center for Biological Evaluation and Research.

Pre-vaccination, samples were defined as seronegative to the different viral antigens if assay results were below the following cut-off values: <150 mIU/mL for measles; <24 ED_50_ (enhanced-PRN assay) and <5 EU/mL (ELISA) for mumps; and <4 IU/mL for rubella. The seronegativity cut-offs evaluated in this study had been determined empirically as part of assay validation and were accepted by the USA Food and Drug Administration (FDA).

Testing to evaluate for sub-optimal response was performed at Day 42. In the case of mumps, this testing was based on the enhanced-PRN assay.^13^ A sub-optimal response was defined as antibody concentrations/titers of measles <200 mIU/mL, mumps <51 ED_50_ (enhanced-PRN assay) and rubella <10 IU/mL. Any child with a sub-optimal response at Day 42 post-vaccination was given the option of being revaccinated. For analyses of antibody responses, data from children who were revaccinated were removed from analysis for timepoints after revaccination.

Post-vaccination seroresponses for MMR vaccine viral antigens in initially seronegative children were defined as antibody concentrations/titers of: ≥ 200 mIU/mL for measles; ≥ 10 EU/mL (PPD ELISA), ≥ 51 ED_50_ (enhanced-PRN assay), or ≥ 4 ED_50_ (unenhanced-PRN assay) for mumps; and ≥ 10 IU/mL for rubella. The seroresponse thresholds evaluated in this study were accepted by the FDA as thresholds defining active immunization offering clinical benefit.

### Safety assessment

An extended safety follow-up assessed SAEs for up to 6 months post-primary vaccination. During this period, we also recorded any new onset chronic illnesses including autoimmune disorders, asthma, type I diabetes, allergies, and conditions prompting emergency department visits. SAEs related to study participation prompting study withdrawal and/or leading to death were recorded for up to 2 y post-vaccination.

### Statistical analysis

The analyses of immunogenicity and safety are descriptive. Antibody persistence was calculated for the ATP cohorts for persistence, which included all eligible children vaccinated with MMR-RIT or M-M-R II, who complied with blood sampling schedules, and who had immunogenicity measurements available for pre-vaccination, Day 42 and Year 2 post-vaccination.

The percentage of children with antibody concentrations ≥ 200mIU/mL (measles), ≥ 10 IU/mL (rubella), ≥ 4 ED_50_ (mumps, by unenhanced-PRN assay) and ≥ 10 EU/mL (mumps, by ELISA) and their exact 95% confidence intervals (CIs) were tabulated for Years 1 and 2 post-vaccination. Post-vaccination antibody GMTs and GMCs were calculated with 95% CIs.

The analysis of the extended safety follow-up and the persistence phase was conducted on the total vaccinated cohort (TVC). The analysis of safety was descriptive. SAEs, new onset chronic illnesses and conditions prompting emergency department visits for up to 6 months post-vaccination were described and reported. Additionally, SAEs related to study participation, study withdrawal and/or leading to death between 6 months post-vaccination and up to 2 y were described and reported.

The statistical analyses were performed using the SAS® software version 9.2 (SAS Institute Inc., Cary, NC, United States) and Proc StatXact 8.1.

## Trademark statement

Priorix is a trademark of GSK group of companies. Proquad is a trademark of Merck and Co., Inc. Enzygnost is a trademark of Dade Behring Marburg GmbH.
